# Künstliche Intelligenz in der Pathologie – wie, wo und warum?

**DOI:** 10.1007/s00292-024-01314-9

**Published:** 2024-03-12

**Authors:** Peter Schüffler, Katja Steiger, Carolin Mogler

**Affiliations:** 1 Institut für Pathologie, TUM School of Medicine and Health, Technische Universität München, München, Deutschland; 2 TUM School of Computation, Information and Technology, Technische Universität München, München, Deutschland; 3 Munich Center for Machine Learning (MCML), München, Deutschland

**Keywords:** Digitale Pathologie, Automatisierung, Digitale Medizin, Qualitätskontrolle, Qualitätssicherung, Digital pathology, Automation, Digital medicine, Quality control, Quality assurance

## Abstract

Künstliche Intelligenz verspricht viele Erneuerungen und Erleichterungen in der Pathologie, wirft jedoch ebenso viele Fragen und Ungewissheiten auf. In diesem Artikel geben wir eine kurze Übersicht über den aktuellen Stand, die bereits erreichten Ziele vorhandener Algorithmen und immer noch ausstehende Herausforderungen.

## Abstract (en)

Artificial intelligence promises many innovations and simplifications in pathology, but also raises just as many questions and uncertainties. In this article, we provide a brief overview of the current status, the goals already achieved by existing algorithms, and the remaining challenges.

Pathologie bricht zurzeit in ein digitales Zeitalter auf, in welcher die Objektträger (OT) eingescannt und digital befundet werden. Digitale Pathologie hat auch bereits ohne künstliche Intelligenz (KI) viele Vorteile. So sind digitale Abbilder der OT leicht miteinander teilbar, was sehr hilfreich ist, da pathologische Expertise nicht unbedingt örtlich gebunden ist. Sie sind schneller auffindbar als physische OT, was bei historischen Fällen die Suche im Archiv oder bei Kollegen obsolet macht. Physische OT kann nur eine Person gleichzeitig in der Hand haben, was bei Konsultationen zu Verzögerungen und mitunter auch zu verlorenen OT führen kann. Digitale OT waschen nie aus und bleiben stets in der gleichen Qualität.

Dennoch ist die Umsetzung einer digitalen Arbeitsweise für ein Institut nicht zu unterschätzen. Die großen Herausforderungen sind hauptsächlich die hohen Anschaffungskosten der benötigten Scanner-Hardware und der nötigen IT-Infrastruktur wie Netzwerk und Speicher, die Einbindung der Digitalisierung in den Laborablauf und die Umstellung auf die Befundung am Bildschirm [1]. Insbesondere die hochauflösenden Pathologiebilder mit einer jeweiligen Größe von 1–2 GB erfordern bei Tausenden Fällen pro Jahr eine durchdachte Strategie, um diese effizient zu speichern und zu streamen. Die digitale Transition ist also keine einfache Aufgabe, aber viele Institute haben ihre Erfahrungen mit individuellen Hürden und Lösungen berichtet, allesamt durchweg erfolgreich [2–5].

Digitale Pathologie verspricht, durch die Möglichkeit der Anwendung von KI im pathologischen Arbeitsablauf die Befundung zu vereinfachen. Das reicht von automatisierten Laborprozessen über Qualitätskontrolle bis hin zur computerunterstützten Krankheitserkennung in digitalen OT (Abb. 1). Insbesondere die automatisierte Bildanalyse profitiert sehr von KI, da eine Kernaufgabe der KI das Erlernen von Mustern ist, und die pathologische Diagnostik ebensolche Muster im Gewebe sucht. Man würde meinen, Pathologie und KI seien wie füreinander geschaffen. Nach anfänglichen Erfolgsgeschichten beispielsweise über automatisierte Krebserkennung in Prostatanadelbiopsien [6] und weiteren Algorithmen [7–9] sind dennoch erstaunlich wenig KI-Anwendungen in der klinischen Pathologie zu finden. Die Entwicklung solcher Algorithmen ist eben sehr zeitaufwändig und bedarf einer gewissen Validierungsphase. Weiterhin benötigt ihre Anwendung im Pathologielabor einige Voraussetzungen wie z. B. die erforderliche technische Infrastruktur und Software. Wir wollen im folgenden Artikel Möglichkeiten und Anwendungsbeispiele von KI (s. Infobox) in der Pathologie erörtern.

**Figure 1:**
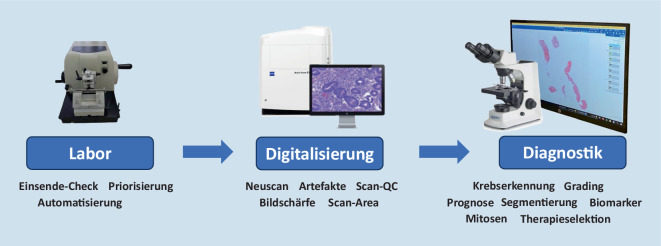

## Infobox Was ist KI?

Laut dem digitalen Wörterbuch der deutschen Sprache der Berlin-Brandenburgischen Akademie der Wissenschaften ist Intelligenz der *„*(Grad der) Fähigkeit, Informationen aufzunehmen, sinnvoll zu verarbeiten und auf dieser Basis rational und kreativ zu handeln*“*. Diese Definition lässt sich gut auf maschinelle Systeme zur KI übertragen. KI-Systeme sollen Information verarbeiten und darauf reagieren und handeln, also Entscheidungen treffen.

## Automatisierte Laborprozesse

Ein Pathologielabor nimmt Frischgewebe (Biopsien, Resektate) an und bereitet es auf, sodass eine Fachärztin oder ein Facharzt das Gewebe optisch oder auch mit molekularpathologischen Methoden untersuchen kann. Diese Aufbereitung besteht im Groben aus der Fallannahme, dem Zuschnitt, der Entwässerung und Fixierung des Gewebes, dem Ausgießen in Paraffin, dem Schneiden und Aufziehen auf OT, der Färbung mit Standardfärbungen, Sonderfärbungen und immunhistochemischen Färbungen, ggf. dem Einscannen der OT und/oder dem Ausliefern der OT an die Ärztinnen und Ärzte.

Automatisierungen haben längst Einzug in das Pathologielabor gehalten. Entwässerungsautomaten entwässern und fixieren die Proben vollautomatisch nach vorgegeben Protokollen. Färbeautomaten übernehmen das standardisierte Eintauchen und Spülen der OT in die Konzentrationsreihen der Färbelösungen. Mehrere Firmen haben sich auf die komplette Laborautomatisierung spezialisiert, bei der alle Schritte vom Einsendegefäß bis zum fertigen Paraffinblock automatisch in einer Roboterstraße durchgeführt werden. Alles, was es dazu braucht, sind Strichkode-markierte Probengefäße.

Vor Anschaffung solcher Geräte würde sicher eine Kosten-Nutzen-Analyse durchgeführt werden, und ggf. ein Test im eigenen Umfeld, um die Eignung aber auch die geforderte Qualität zu verifizieren. Aber grundsätzlich hat sich gezeigt, dass solche Geräte für eine gleichbleibende Qualität im Labor hilfreich sind, und dass sie es dem Laborpersonal erleichtern, den Tagesdurchsatz an eingesendetem Material effizient zu bewältigen.

Die Automatisierung im Laborworkflow kann durchaus weitergedacht werden. So könnten mit Hilfe optischer Verfahren Makrosegmente automatisch vermessen und gezählt werden, um bereits Probencontainer vorzubereiten. Gewebefragmente könnten dann mit dem Einsendeschein verglichen werden, um die richtige Patientenzuordnung zu sichern. Befundtemplates könnten so bereits automatisch angelegt werden, die in einer einheitlichen Struktur der Anzahl der Gewebefragmente in den Einsendegefäßen entsprechen. Die Einsendungen könnten weiterhin bereits im Eingang priorisiert und den Ärztinnen und Ärzten der Expertise und Verfügbarkeit nach zugeordnet werden. Ähnlich wie die bisherigen automatisierten Prozesse wären auch diese erweiterten Laboraktionen hilfreiche Unterstützung, vorausgesetzt sie funktionieren reibungslos.

## KI zur Qualitätskontrolle der OT

Die Digitalisierung der OT ist ein zusätzlicher Schritt im Labor. Hochauflösende Whole-slide-Scanner generieren dabei digitale Bilder der OT bei 200facher oder 400facher Vergrößerung, äquivalent zu gebräuchlichen Lichtmikroskopen. Dieser Prozess dauert zwar nur wenige Minuten pro OT, aber größere Fälle mit dutzenden OT benötigen dadurch 1–2 h, bis sie fertig sind – je nachdem, wie viele OT in der Warteschlange sind. Eine Parallelisierung des Scannens ist zwar möglich, bedarf aber die nötige Investition in mehrere Scannergeräte [5].

Digitale OT sind statische Momentaufnahmen. Das gescannte Areal, die Tiefenschärfe, Artefakte und vieles mehr sind nach dem Scan im Bild nicht mehr änderbar. Daher sind qualitativ hochwertige Scanner unerlässlich, die bei allen Variationen von Geweben und OT präzise Bilder generieren.

Das vollständige Scannen des ganzen Gewebes auf dem OT ist essenziell, da ungescanntes Gewebe der Pathologin oder dem Pathologen später nicht mehr zur Befundung zur Verfügung steht. Das automatische Erkennen des Gewebes kann aber insbesondere bei blassen Färbungen und kleinen, fragmentierten Präparaten durchaus eine Herausforderung sein. Fremdkörper wie Wachsreste, Staub und Fingerabdrücke erschweren den Prozess weiter. KI-basierte Algorithmen können hier helfen [10].

Eine gute Bildschärfe ist für digitale Pathologie entscheidend. Unschärfe entsteht, wenn das Bildmaterial nicht im Fokuspunkt der Linse liegt. Das passiert z. B. bei ungleichmäßig dick geschnittenen Geweben wie z. B. Proben von Knochen, Faltungen oder Risse im Gewebe während der Herstellung der OT, eingeschlossenen Luftblasen oder Fremdkörpern oder bei losen Zellstrukturen wie bei Zytologieproben [11]. Die Erkennung von unscharfen Bildarealen mithilfe von KI ist Gegenstand aktueller Forschung [12–14]. Zwar existieren bereits einige offene und proprietäre Algorithmen, ihre Rückkopplung an den Scanningprozess ist allerdings bisher nicht adressiert.

Artefakte sind Störungen im digitalen Bild, welche die Interpretation des Bildes erschweren. Sie können bereits physisch auf dem OT oder durch den Digitalisierungsprozess entstanden sein. Physische Artefakte sind Gewebefaltungen und -risse, eingeschlossene Luftblasen, ein fehlendes Deckglas oder Risse im Deckglas, Stiftmarkierungen oder Fremdkörper. Digitale Artefakte sind z. B. die oben angesprochene Unschärfe, nicht erkanntes und damit ungescanntes Gewebe oder Bildstreifen durch einen missglückten Weißabgleich oder eine verschmutzte Linse.

Die Qualitätskontrolle in digitalen Pathologiebildern mit Methoden der KI ist ein wichtiges Feld der computergestützten Pathologie [11, 15]. Es gibt deutliche Fortschritte in der Erkennung vieler der genannten Artefakte, jedoch nur wenig Möglichkeiten, damit umzugehen. Ein automatisiertes Auslösen eines Neuscans wäre denkbar sowie das Aussortieren des betroffenen OT zum neuerlichen Schneiden (etwa bei größeren Falten oder fehlendem Deckglas). Bisher wird das erst später im Workflow entschieden, nämlich wenn die Pathologin oder der Pathologe den OT nicht interpretieren kann.

## KI-unterstützte Auswertung und Befundung

Die eigentliche Bildinterpretation beherbergt wohl das größte Potenzial von KI in der Pathologie. Zahlreiche Algorithmen wurden bereits entwickelt und erforscht, um den diagnostischen Prozess zu unterstützen. Dabei wird dieser Prozess in Einzelschritte wie Detektion, Grading, Quantifizierung, Subtypisierung, Biomarkererkennung, Prognose und Therapieantwort unterteilt [16]. Für jeden Schritt existieren spezifische Algorithmen, jeweils auf eine Krankheitsart fokussiert, welche diesen Schritt unterstützen. Diese Algorithmen basieren je nach Studie auf digitalen Hämatoxylin-Eosin (HE)-gefärbten oder immunhistochemisch (IHC-)gefärbten Proben.

Ein Beispiel für Detektion ist das Modell von Campanella et al. [6], welches Prostatakrebs in Nadelbiopsien mit einer „area under the curve“ (AUC) von 0,99 erkennt (ungeachtet des Grades). Ihr Modell ließ sich ohne Weiteres auch auf andere Krebsarten anwenden (Basalzellenkrebserkennung, AUC = 0,988 und Lymphknotenmetastasenerkennung, AUC = 0,966).

Ström et al. [7] und Bulten et al. [8] haben je ein Modell entwickelt und validiert, das weitere diagnostische Informationen ausgibt. Ströms Modell konnte nicht nur mit einer AUC von 0,986 den Krebs erkennen, sondern auch mit einer Korrelation von 0,87 (zum manuellen Messen) dessen Länge quantifizieren und mit einem Kappa von 0,62 den Gleason Score beurteilen, etwa vergleichbar mit dem Inter-Pathologen-Kappa von 0,60–0,73. Bulten et al. [8] zeigten, dass die Übereinstimmung des Gleason Scores von Pathologen, welche die KI-Assistenz nutzten, besser war als bei Pathologen ohne KI (Cohen’s Kappa 0,786 vs. 0,733), und übrigens auch besser als bei KI allein.

Cireşan et al. [17] veröffentlichten bereits 2013 einen Algorithmus zur Quantifizierung von Mitosen in digitaler Brusthistologie. Van Bergeijk et al. [18] haben diesen Algorithmus 10 Jahre später in einer Studie validiert mit dem Ergebnis, dass Pathologen, welche die KI-Assistenz nutzen, eine höhere Korrelation mit den im Lichtmikroskop gesehenen Mitosen aufweisen als die, die die KI-Assistenz nicht nutzen.

Die KI-unterstützte Subtypisierung ist ein großes Feld. Prominente Beispiele sind die Bestimmung vom Östrogenrezeptor (ER), dem Progesteronrezeptor (PR) und dem humanen epidermalen Wachstumsfaktor (Her2). Naik et al. [19] konnten 2020 mit einer AUC von 0,92 den ER-Status in HE-Proben von Brustkrebspatientinnen mittels eines gelernten Algorithmus erkennen. Ein Jahr später haben Gamble et al. [20] eine KI entwickelt, die zusätzlich zum ER-Status auch den PR-Status und den Her2-Status erkennt, mit einer AUC von 0,86, 0,75 und 0,60. Kather et al. [21] konnten gar genetische Mikrosatelliteninstabilität (MSI) im HE-Bildern von kolorektalen Karzinomen detektieren, was mit einer AUC von 0,84 bereits sehr verlässlich ging. Man sieht in diesen Beispielen deutlich, dass die Forschung zwar vielversprechende Erfolge erzielt, aber für eine klinische Anwendung noch weitere Verbesserungen nötig sind.

### KI zur Priorisierung und Qualitätssicherung

Wie in den obigen Beispielen skizziert, können bildverarbeitende Algorithmen tatsächlich Muster in der Morphologie erkennen und daraus relevante Informationen extrahieren, manche davon bereits so genau, dass sie in den Befundungsprozess einbezogen werden können. So könnten Fälle bereits vor Befundung automatisch nach Präsenz und Grad eines malignen Tumors priorisiert werden, um diese Fälle rasch einer Pathologin oder einem Pathologen zu präsentieren und den Befund und den Therapiebeginn ggf. zu beschleunigen. Weiter könnte KI zur Qualitätssicherung genutzt werden: In zahlreichen Studien [8, 22, 23] wurde bereits gezeigt, dass insbesondere das Zusammenspiel zwischen Pathologin oder Pathologe und KI-Modell jeweils die genauesten Ergebnisse erzielte. Das ist in etwa vergleichbar mit pathologischen Zweitmeinungen oder Konsensusbefunden, bei denen mehrere Pathologinnen oder Pathologen einen Fall bewerten und so zu einem sichereren Ergebnis kommen. KI könnte also als systematische Zweitmeinung bei Krebsfällen die manuelle Interpretation bestätigen oder bei Diskrepanzen gezielt darauf hinweisen und zur Reflexion anregen. Schließlich ist eine Zeitersparnis bei der Befundung denkbar, indem der Algorithmus einen relevanten Hotspot direkt aufzeigt, was insbesondere bei kleinen Läsionen oder einzelnen Zellen eine manuelle Suche ersparen kann. Zeitersparnis wurde allerdings erst im Hinblick auf digitale Pathologie ohne KI tiefer untersucht [24–26], und verlässliche Erfahrungswerte mit KI-Nutzung sind ausstehend.

### KI als virtueller Biomarkertest

Erstaunlicherweise können Modelle auch Information aus HE-Bildern extrahieren, welche bisher sonst mit IHC- oder molekularpathologischen Tests gewonnen werden müssen. Beispiele sind die oben beschriebenen ER-, PR- und Her2-Statusmodelle [19, 20] oder das MSI-Modell [21]. KI kann sich also auch zum Aufspüren neuer virtueller Biomarker in HE-Proben eignen. Sollten solche virtuellen Tests für einzelne Biomarker klinisch verfügbar sein, wäre das ein drastischer Zeitgewinn, da sie im Gegensatz zu molekularen Tests innerhalb von Minuten das Ergebnis präsentieren. Auch wären sie als virtuelle Tests mit deutlich niedrigeren Kosten verbunden.

### Welche Modelle sind klinisch verfügbar?

Mit der Pathologie-KI verhält es sich ähnlich wie in der restlichen klinischen Forschung: Aus vielen Forschungsansätzen resultieren wenige klinische Produkte. Viele Hypothesen in der Forschung halten einer externen Validierung nicht Stand, möglicherweise wegen der hohen Variabilität des Gewebes, verschiedenartiger Laborprotokolle oder wegen fehlender Standards in der Digitalisierung (Stichwort *Scannervariabilität*) oder sie sind schlicht nicht genau genug für klinische Anwendung. Die Zulassung von KI in der Pathologie ist für die regulatorischen Behörden zudem ein neues Feld. Die Food and Drug Administration (FDA) hat 2021 das erste KI-basierte Modell in der Pathologie überhaupt zugelassen und dabei neue Richtlinien für diese Kategorie geschaffen [27]. Die Anzahl der FDA-zugelassenen computerbasierten Modelle in der Radiologie ist 531, während es in der Pathologie gerade mal 4 sind, und nur eins davon ist rein bildbasiert (Stand Feb 2024)^1^. Dennoch können wir bei dem bisherigen Wachstum in der Forschung davon ausgehen, dass mittelfristig immer mehr Algorithmen klinisch verfügbar sind und sich das Angebot deutlich vergrößert. Die Anzahl der Publikationen im Feld wächst zumindest zurzeit exponentiell [28].

## Alles aus vielen Händen

Es herrscht eine gewisse Entrepreneur-Stimmung im Feld der Pathologie-KI. Viele bekannte Firmen sind Ausgründungen aus den jeweiligen Universitäten oder Universitätsspitälern und nicht etwa neue Teile von großen, etablierten Laborausstattern, Pathologie- oder Pharmakonzernen. Das führt auch zu einer Vielzahl von neuen Programmen und Software, die in den jeweiligen Firmen entwickelt wird, angefangen von ihren Kernprogrammen, bis hin zu Arbeitslisten und Viewern, in denen ihre Algorithmen und die digitalen Bilder benutzerfreundlich visualisiert werden.

Eine Herausforderung dabei ist, dass Pathologien bereits mit Laborinformationssystemen (LIS) arbeiten, welche die Aufträge, Laborleistungen, Fälle, Befunde, Abrechnungen u. v. m. organisieren. Diese Programme sind oftmals alt, komplex gewachsen und brauchen neue Schnittstellen, um mit der digitalen Pathologie, den Scannern, den Imagemanagementsystemen und den KI-Systemen zu kommunizieren. Andernfalls müssten die Benutzerinnen und Benutzer ständig die Software wechseln, was im Pathologiealltag nicht effizient ist.

Ein weiteres mögliches Problem ist die Zersplitterung der vielen Anbieter einzelner Modelle. Muss ein Labor, welches umfangreiche KI nutzen will, verschiedene Systeme anschaffen, die allesamt an das LIS angepasst werden müssen, mit verschiedenen Viewern und Interfaces kommen und höchstwahrscheinlich separate Speicherorte der digitalen Bilder nutzen? Hier wäre eine Lösung, eine Art Standard für KI zu entwickeln, mit welchen Inputs und Outputs herstelleragnostisch prozessiert werden können. So könnte ein Institut sich unabhängig vom Viewer für verschiedene KI-Lösungen entscheiden und sie einfach in den Workflow je nach Bedarf einpflegen und kombinieren.

## Fazit für die Praxis

- Pathologie-KI (künstliche Intelligenz) verbindet Pathologie mit Informatik, für erweiterte Automatisierung im Labor, verbesserte Digitalisierung, unterstützte Befundung, systematische Qualitätssicherung und neue, virtuelle Biomarker.
- Eine interaktive und intensive Zusammenarbeit zwischen Pathologischen und Informatischen Fachkräften ist unabdingbar, um die nächsten Algorithmen der künstlichen Intelligenz synergetisch zu entwickeln.
- Viele Algorithmen existieren bereits zur Beantwortung einzelner Fragestellungen und werden über die Zeit verbessert und weiter validiert, um schließlich in die klinische Anwendung zu kommen. Dabei hat sich gezeigt, dass die Algorithmen Pathologinnen und Pathologen durchaus unterstützen: Das Zusammenspiel zwischen Mensch und Maschine erzielt in zahlreichen Studien bessere Ergebnisse als sie jeweils alleine erreichen.
- Pathologie-KI erweist sich demnach als brauchbares und spannendes Tool, das man nicht unterschätzen sollte.

## Footnotes

1 https://www.fda.gov/medical-devices/software-medical-device-samd/artificial-intelligence-and-machine-learning-aiml-enabled-medical-devices.
